# Responses of host energy status, intestinal structure and gut microbiota during post-hibernation recovery in high- and low-altitude populations of the plateau frog *Rana kukunoris*

**DOI:** 10.3389/fphys.2026.1891419

**Published:** 2026-07-27

**Authors:** Huihui Wang, Lu Jiang, Liye Zhong, Haoqi Zhang, Yajie Li, Zhaoxuan Zhai, Wenying Liu, Miaojun Ma, Qiang Chen, Xiaolong Tang

**Affiliations:** 1 School of Stomatology, Lanzhou University, Lanzhou, Gansu, China; 2 Department of Animal and Biomedical Sciences, School of Life Sciences, Lanzhou University, Lanzhou, Gansu, China; 3 State Key Laboratory of Grassland and Agro-Ecosystems, College of Ecology, Lanzhou University, Lanzhou, Gansu, China

**Keywords:** gut microbiota, high-altitude adaptation, intestinal histology, metabolic rate, metagenomics, post-hibernation recovery, *Rana kukunoris*

## Abstract

**Background:**

Hibernation is an important seasonal strategy that enables amphibians to cope with low temperature and food scarcity. However, how high- and low-altitude amphibian populations differ in host energy status, digestive system structure and gut microbiota during post-hibernation recovery remains insufficiently understood.

**Objective:**

This study aimed to evaluate post-hibernation changes in host energy status, intestinal structure and gut microbial composition and functional potential in high- and low-altitude populations of the plateau frog *Rana kukunoris*.

**Methods:**

We compared high-altitude and low-altitude populations of *R. kukunoris* before and after hibernation by integrating morphological traits, whole-animal metabolic rate, digestive tract length, small-intestinal histology and shotgun metagenomic profiles of small-intestinal contents.

**Results:**

After hibernation, both populations showed significant decreases in body mass, liver mass and hepatosomatic index, together with increased whole-animal metabolic rate, indicating a transition from energy reserve depletion to metabolic recovery. The hepatosomatic index showed a significant altitude × stage interaction, suggesting stronger relative liver energy depletion in the low-altitude population. Digestive system analysis showed that the low-altitude population exhibited more pronounced structural remodeling, including shortened digestive tract length, increased muscularis thickness and reduced epithelial thickness after hibernation, whereas the high-altitude population showed a relatively conservative response. Metagenomic analysis showed that alpha diversity remained relatively stable, whereas beta diversity, dominant microbial taxa and functional potential shifted among groups. Microbial functional profiles were mainly associated with metabolism, nutrient transformation and carbohydrate utilization.

**Conclusion:**

Post-hibernation recovery in *R. kukunoris* involves cross-level parallel responses in host energy status, digestive system structure and gut microbiota. High- and low-altitude populations may adopt different physiological recovery strategies after hibernation, providing new evidence for understanding seasonal adaptation in amphibians inhabiting cold environments.

## 1 Introduction

Cold high-altitude environments are characterized by low temperature, hypoxia, strong ultraviolet radiation and short activity seasons, which impose persistent pressures on survival, growth, reproduction and energy allocation in ectothermic animals. Amphibians are particularly sensitive to environmental temperature and seasonal resource availability because their body temperature and metabolic activity largely depend on external conditions. They therefore provide useful models for studying high-altitude adaptation and low-temperature physiological regulation. Previous studies have shown that high-altitude frogs exhibit adaptive changes at multiple biological levels, including genome-level adaptive evolution (Chen et al., 2023), transcriptomic signals related to energy metabolism (Yang et al., 2016), physiological and biochemical adjustments in antioxidant defense and energy metabolism (Niu et al., 2022; Tang et al., 2026), and changes in host metabolism and gut microbial function (Niu et al., 2025). In addition, behavioral and physiological traits of frogs can be adjusted under low-temperature conditions, further suggesting that environmental temperature is an important driver of adaptive divergence in amphibians (Muir et al., 2014). The plateau frog *R. kukunoris*, which is distributed across the Qinghai-Tibet Plateau and surrounding regions, has been shown to possess high-altitude adaptive features in genome structure and metabolic regulation (Chen et al., 2023; Niu et al., 2025; Tang et al., 2026), making it a suitable model for investigating seasonal adaptation in amphibians inhabiting cold environments.

Hibernation is an important seasonal strategy that allows amphibians to cope with prolonged low temperature and food scarcity. It is characterized by reduced activity, metabolic depression and mobilization of endogenous energy reserves. Previous studies on amphibian hibernation have mainly focused on metabolic depression during overwintering, reduced aerobic capacity of tissues and changes in immune status (Cooper et al., 1992; St‐Pierre and Boutilier, 2001). However, the post-hibernation recovery period is also physiologically important. During this stage, animals need to resume activity, restart feeding, compensate for energy loss during overwintering and prepare for subsequent reproduction and growth. Recent studies on frogs during hibernation and spring emergence indicate that post-hibernation individuals do not simply return to their pre-hibernation state. Instead, they undergo a dynamic transition from energy depletion to physiological recovery, during which gut microbial diversity, composition and functional potential may also change (Tong et al., 2023; Gorokhova et al., 2024). Thus, post-hibernation recovery represents an important window for understanding seasonal physiological regulation and environmental adaptation in amphibians.

The digestive system is a key organ system for restoring feeding, rebuilding energy balance and compensating for overwintering energy depletion after hibernation. Its morphology and histological organization can reflect digestive and absorptive capacity, organ plasticity and energy allocation strategies. Comparative physiological studies have shown that the intestines of amphibians and reptiles exhibit pronounced reversible regulation in response to feeding status, fasting and refeeding, including changes in intestinal mass, epithelial structure, digestive enzyme activity and nutrient transport capacity (Secor, 2005a). In the model amphibian *Xenopus laevis*, fasting and refeeding induce substantial changes in intestinal morphology and digestive enzyme activity, together with systematic transcriptional and epigenetic responses (Tamaoki et al., 2016). Therefore, examining digestive tract length and small-intestinal histological structure before and after hibernation may help clarify digestive system plasticity and its potential energetic significance during seasonal transitions in *R. kukunoris*.

Gut microbiota represents an important symbiotic system involved in nutrient degradation, energy acquisition, immune regulation and environmental adaptation. Studies in amphibians have shown that artificial hibernation, natural overwintering, seasonal transitions, habitat differences and environmental microbial exposure can all affect gut microbial community structure and functional potential (Bletz et al., 2016; Weng et al., 2016; Tong et al., 2019; Zhou et al., 2020; Emerson and Woodley, 2024). For example, gut microbial composition and predicted functions are markedly restructured during artificial hibernation in the brown tree frog *Polypedates megacephalus* (Weng et al., 2016). In *Rana dybowskii*, seasonal hibernation alters the composition and similarity of skin and gut microbiota (Tong et al., 2019). Across different habitats, amphibian gut microbial communities may diverge in taxonomic structure while converging on habitat-specific functional profiles (Bletz et al., 2016). These findings suggest that gut microbiota may participate in nutrient transformation and energy utilization during seasonal low temperature, fasting and post-hibernation feeding recovery.

From a broader host–microbiome perspective, gut microbiota are not merely passive communities inhabiting the digestive tract, but may also contribute to host adaptation to environmental change. Theories of host–microbe coevolution and the holobiont/hologenome concept propose that hosts and their associated microbiota can function as integrated biological units that jointly respond to environmental pressures and contribute to host phenotypes, metabolic regulation, immune homeostasis and ecological adaptation (Ley et al., 2006; Rosenberg and Zilber‐Rosenberg, 2011; Shapira, 2016; Rosenberg and Zilber-Rosenberg, 2018). Recent reviews on hibernator microbiomes further emphasize that prolonged fasting, bile and mucus recycling, metabolic depression and rapid post-emergence recovery are commonly accompanied by gut microbial restructuring and functional shifts (Gao et al., 2024). However, in amphibians, particularly under high-altitude conditions, it remains unclear how host energy status, digestive system structure and gut microbiota change together during post-hibernation recovery.

To address this gap, we investigated high- and low-altitude populations of *R. kukunoris* from Maqu and Minhe, respectively. Four groups were defined according to altitude and hibernation stage: pre-hibernation high-altitude group (PH-HA), after-hibernation high-altitude group (AH-HA), pre-hibernation low-altitude group (PH-LA) and after-hibernation low-altitude group (AH-LA). We compared body mass, liver mass, hepatosomatic index, whole-animal metabolic rate, digestive tract length, small-intestinal histology and metagenomic profiles of small-intestinal contents among these groups. This study aimed to systematically evaluate changes in host energy status, digestive system structure, gut microbial composition and microbial functional potential during post-hibernation recovery in *R. kukunoris* from different altitudes. Based on previous evidence that amphibian hibernation involves prolonged fasting, mobilization of energy reserves, metabolic depression and subsequent metabolic reactivation after emergence, we hypothesized that post-hibernation frogs would show concurrent energy depletion and metabolic recovery. In light of altitude-related physiological plasticity and potential differences in seasonal recovery strategies, we further hypothesized that the low-altitude population would exhibit stronger digestive structural remodeling and metabolic activation than the high-altitude population. Finally, we hypothesized that altitude and hibernation stage would be associated with shifts in gut microbial composition and functional potential, particularly in microbial functions related to nutrient transformation and carbohydrate utilization, showing group-level parallel responses with host recovery.

## 2 Materials and methods

### 2.1 Animal collection and grouping

**Figure 1:**
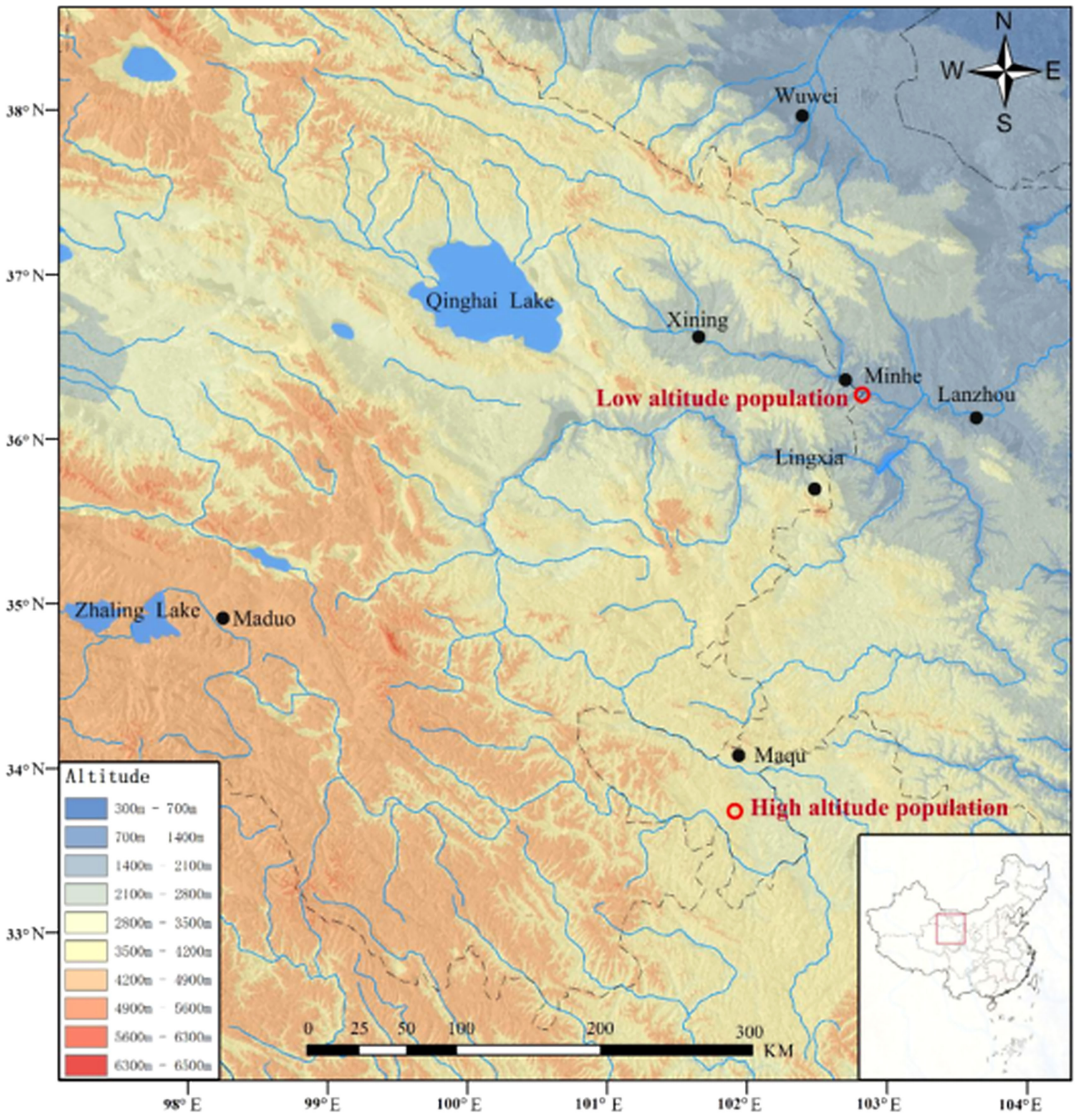
Sampling sites of high- and low-altitude populations of *Rana kukunoris*. Red circles indicate the sampling sites in Maqu County, Gansu Province, and Minhe Hui and Tu Autonomous County, Qinghai Province, China.

Adult male plateau frogs (*R. kukunoris*) were used in this study. Two geographical populations from different altitudes were selected for comparison. The high-altitude population was collected from Maqu County, Gansu Province, China (101.88°E, 33.67°N; 3468.79 m above sea level), and the low-altitude population was collected from Minhe Hui and Tu Autonomous County, Qinghai Province, China (102.76°E, 36.20°N; 2052.20 m above sea level) (**Figure 1**).

Pre-hibernation samples were collected in October, and after-hibernation samples were collected in May. According to sampling altitude and hibernation stage, the frogs were assigned to four groups: PH-HA, AH-HA, PH-LA and AH-LA. The collected individuals were used for morphological measurements, whole-animal metabolic rate analysis, digestive tract length measurement, small-intestinal histology and metagenomic sequencing of small-intestinal contents. All animal procedures were approved by the Institutional Animal Care and Use Committee of School of Stomatology Lanzhou university (approval number: LZUKQ-2023-013) and were conducted in accordance with institutional and national guidelines for the care and use of experimental animals. All efforts were made to minimize animal suffering and reduce the number of animals used.

### 2.2 Morphological measurements, digestive tract length and metabolic rate

Morphological measurements were performed on field-collected adult male frogs. Adult male frogs were identified based on external morphology, body size and gonadal development confirmed during dissection. Snout–vent length (SVL) was measured to the nearest 0.01 cm using a digital caliper. Body mass was measured to the nearest 0.01 g using an electronic balance, and liver mass was measured to the nearest 0.0001 g using an analytical balance. The hepatosomatic index (HSI) was calculated as liver mass/body mass × 100%. After dissection, the entire digestive tract was removed and gently laid flat on coordinate paper, and digestive tract length was measured from the stomach to the cloaca. Whole-animal metabolic rate was measured at 10 °C. This temperature was selected because previous field observations showed that the water temperature during the active season of *R. kukunoris* was approximately 10 °C. Before measurement, frogs were acclimated at 10 °C and fasted for 48 h to minimize the influence of feeding status on metabolic measurements. CO_2_ production was measured using an animal respirometry system (Sable Systems, USA). Body mass was recorded before measurement. Mass-specific metabolic rate was calculated based on the difference in CO_2_ concentration between incurrent and excurrent air, flow rate, measurement temperature and barometric pressure, and expressed as mg CO_2_ min^−1^ g^−1^.


$$\text{Metabolic rate}=\frac{\frac{\text{M}({\text{CO}}_{2})}{22.4}\times {\text{Cco}}_{2}(\text{ppm})\times \frac{273}{272+\text{T}}\times \frac{\text{P}}{{\text{P}}_{0}}\times \text{flow rate}}{\text{body mass}}$$

### 2.3 Small-intestinal histology

Small-intestinal tissues were immediately fixed in 4% paraformaldehyde for histological analysis. Fixed tissues were rinsed with PBS and then processed using standard paraffin-embedding procedures, including dehydration, clearing, paraffin infiltration, embedding and sectioning. Sections were stained with hematoxylin and eosin (H&E), observed under a light microscope and photographed.

Histological parameters were measured using ImageJ software, including lumen diameter, muscularis thickness, villus height and epithelial thickness. For each sample, well-oriented sections and fields with intact structures were selected, and the mean value was used for subsequent statistical analysis.

### 2.4 Metagenomic sequencing and bioinformatic analysis of small-intestinal contents

Small-intestinal contents were collected under sterile conditions and immediately frozen in liquid nitrogen before storage at −80 °C. Four small-intestinal content samples from each group were randomly selected for metagenomic sequencing. Microbial genomic DNA was extracted from small-intestinal contents, and DNA integrity and quality were assessed. Qualified DNA samples were randomly fragmented to approximately 350 bp using a Covaris M220 system for library construction. Sequencing was performed on an Illumina NovaSeq platform (Illumina, USA) by Shanghai Majorbio Bio-pharm Technology Co., Ltd.Raw sequencing data were processed using the shotgun metagenomic workflow on the Majorbio Cloud Platform (https://cloud.majorbio.com/). Raw reads were quality-filtered to remove adapter sequences, low-quality reads and host-derived sequences, generating clean reads. Clean reads were then assembled, and open reading frames (ORFs) were predicted. Predicted ORFs were subjected to redundancy removal to construct a non-redundant gene catalog. Clean reads from each sample were mapped back to the non-redundant gene catalog to calculate gene abundance. Taxonomic annotation was performed on the Majorbio Cloud Platform by aligning non-redundant gene sequences against the NR database using DIAMOND. Functional annotation was performed against COG, KEGG and CAZy databases to characterize microbial functional potential.

Community composition and relative abundance were summarized at different taxonomic levels, with a focus on dominant taxa at the phylum and genus levels. Alpha diversity was assessed using ACE, Shannon and Simpson indices were calculated using the metagenomic diversity analysis module on the Majorbio Cloud Platform. Beta diversity was evaluated using the metagenomic diversity analysis module on the Majorbio Cloud Platform by principal coordinate analysis (PCoA) based on Binary Jaccard and Bray–Curtis distance matrices. Differences in community structure among groups were assessed using ANOSIM with 999 permutations. Differentially enriched taxa were identified using linear discriminant analysis effect size analysis. Taxa with P<0.05 and an LDA score>2.0 were considered significantly enriched.

### 2.5 Statistical analysis

Data are presented as mean ± standard error (mean ± SE). Statistical analyses were performed using SPSS 20.0 and GraphPad Prism 9.0.For body mass, liver mass, HSI, metabolic rate, digestive tract length and histological parameters, outliers were first identified and removed using the within-group IQR method (1.5 × IQR).

Planned pairwise comparisons included PH-HA vs AH-HA, PH-LA vs AH-LA, PH-HA vs PH-LA and AH-HA vs AH-LA. Homogeneity of variance was assessed using Levene’s test before pairwise comparisons. When Levene’s test showed *P*>0.05, an independent-samples t-test assuming equal variance was used. When Levene’s test showed *P* < 0.05, Welch’s corrected t-test was used. *P* < 0.05 was considered statistically significant.

Two-way ANOVA was performed to evaluate the effects of altitude, hibernation stage and their interaction on host energy status, metabolic rate, digestive tract length and small-intestinal histological parameters. Altitude included HA and LA, Stage included PH and AH, and Altitude × Stage indicated the interaction between altitude and hibernation stage. Two-way ANOVA was also performed after outlier removal using the within-group IQR method. *P* < 0.05 was considered statistically significant.

Microbial alpha diversity was evaluated using ACE, Shannon and Simpson indices, and group differences were analyzed using the Kruskal–Wallis test. Beta diversity was calculated based on Binary Jaccard and Bray–Curtis distance matrices and visualized using PCoA. Differences in microbial community structure among groups were evaluated using ANOSIM. Microbial community composition was summarized based on relative abundance at different taxonomic levels, mainly at the phylum and genus levels. Differentially enriched taxa were identified using LEfSe analysis. Functional annotation was performed based on COG, KEGG and CAZy databases, and the relative abundance distributions of functional categories, pathways and enzyme classes were summarized.

## 3 Results

### 3.1 Changes in host energy status and metabolic rate

**Figure 2:**
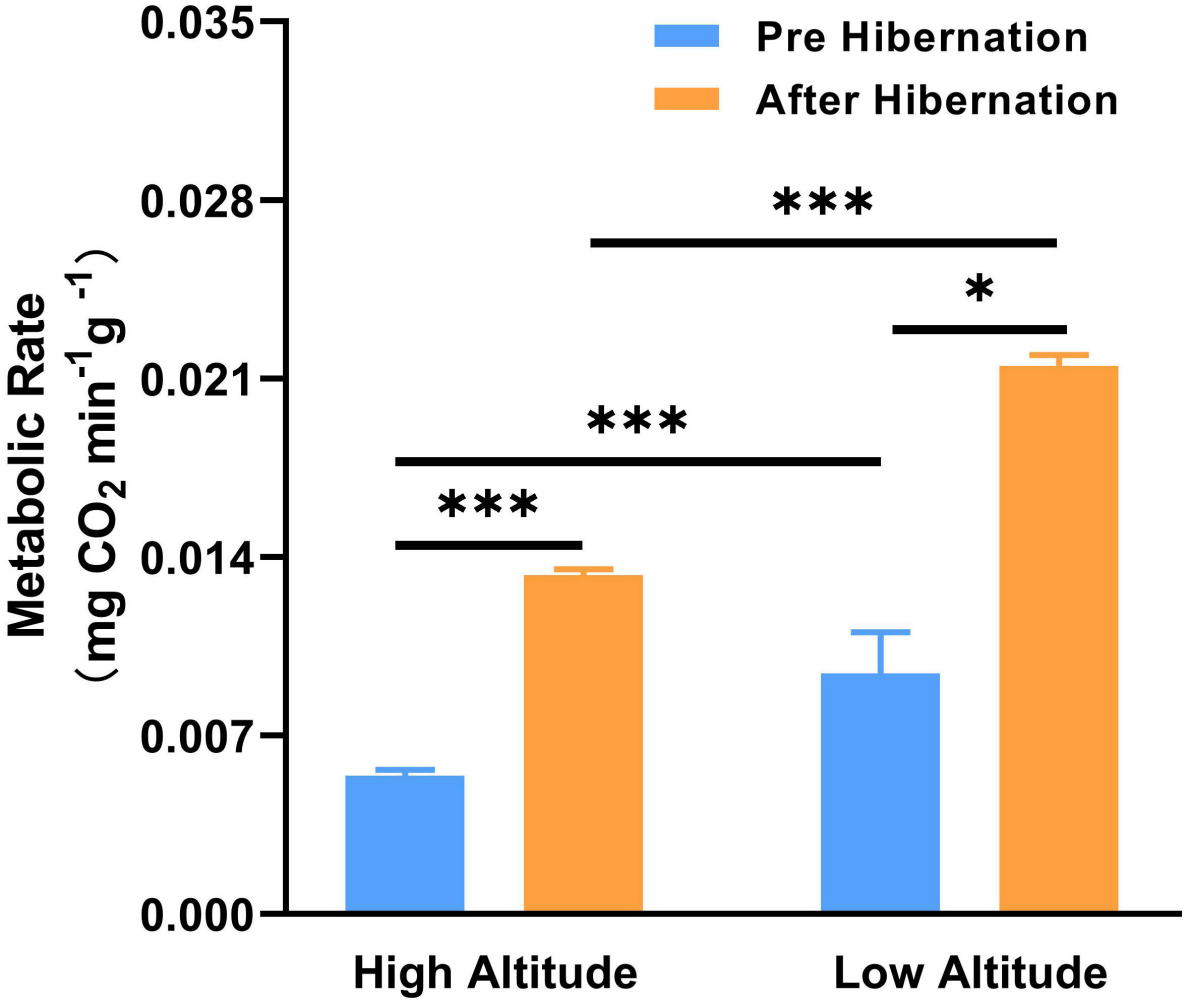
Changes in metabolic rate before and after hibernation in high- and low-altitude populations of *Rana kukunoris*. Data are presented as mean ± SE. * stands for P ≤ 0.05, *** stands for P < 0.001.

To evaluate changes in host energy status and metabolic level before and after hibernation in high- and low-altitude populations of *R. kukunoris*, body mass, liver mass, HSI and whole-animal metabolic rate at 10 °C were analyzed using planned pairwise comparisons and two-way ANOVA (**Tables 1**, **2**; **Figure 2**). Overall, both populations showed decreased body mass, liver mass and HSI after hibernation, whereas metabolic rate increased, indicating a transition from energy depletion to metabolic recovery.

**Table 1:** Comparison of morphological data and metabolic rate between high-and low-altitude populations of *R. kukunoris* before and after hibernation.

Group	Body mass (g)	Snout-vent length (cm)	Liver mass (g)	HSI(%)	Metabolic rate (mg CO_2_ min^−1^g-1)
PH-HA	12.84 ± 0.57^†^(n=14)	5.12 ± 0.07^†^(n=14)	1.08 ± 0.09^†^(n=15)	7.81 ± 0.25^†^ (n=11)	0.0054 ± 0.0002#^†^ (n=5)
AH-HA	9.90 ± 0.47*^†^ (n=14)	4.71 ± 0.11^†^ (n=14)	0.40 ± 0.03*^†^ (n=15)	3.78 ± 0.16*^†^ (n=15)	0.0133 ± 0.0002*^†^ (n=4)
PH-LA	11.22 ± 0.73 (n=16)‡	4.88 ± 0.10(n=17)	0.89 ± 0.0` 3‡ (n=13)	8.31 ± 0.28‡ (n=15)	0.0094 ± 0.0016#‡ (n=8)
AH-LA	6.85 ± 0.16*‡ (n=14)	4.84 ± 0.06(n=14)	0.12 ± 0.01*‡ (n=15)	1.60 ± 0.05*‡ (n=14)	0.0215 ± 0.0004*‡ (n=6)

Data are presented as mean ± standard error of the mean (mean ± SE). * indicates a significant difference between AH-HA and AH-LA (*P* < 0.05); ^#^ indicates a significant difference between PH-HA and PH-LA (*P* < 0.05); ^†^ indicates a significant difference between PH-HA and AH-HA (*P* < 0.05); ^‡^ indicates a significant difference between PH-LA and AH-LA (*P* < 0.05).

**Table 2:** Two-way ANOVA results for host energy status and metabolic rate.

Variable	Effect	df	*F*	*P*
Body mass	Altitude	1, 54	17.939	<0.001
	Stage	1, 54	45.377	<0.001
	Altitude × Stage	1, 54	1.749	0.192
Liver mass	Altitude	1, 55	19.555	<0.001
	Stage	1, 55	187.237	<0.001
	Altitude × Stage	1, 55	0.798	0.375
HSI	Altitude	1, 52	22.828	<0.001
	Stage	1, 52	766.336	<0.001
	Altitude × Stage	1, 52	45.728	<0.001
Metabolic rate	Altitude	1, 19	23.212	<0.001
	Stage	1, 19	75.894	<0.001
	Altitude × Stage	1, 19	2.947	0.102

Altitude indicates sampling altitude, Stage indicates hibernation stage, and Altitude × Stage indicates their interaction.

Body mass was significantly affected by altitude and stage (Altitude: *F_1,54_* = 17.939, *P* < 0.001; Stage: *F_1,54_* = 45.377, *P* < 0.001), whereas the altitude × stage interaction was not significant (*F_1,54_* = 1.749, *P* = 0.192). Planned comparisons showed that body mass was significantly lower in AH-HA than in PH-HA (*P* < 0.001) and significantly lower in AH-LA than in PH-LA (*P* < 0.001). No significant difference was detected between PH-HA and PH-LA (*P* = 0.100), whereas AH-LA had significantly lower body mass than AH-HA (*P* < 0.001).

Liver mass was also significantly affected by altitude and stage (Altitude: *F_1,55_* = 19.555, *P* < 0.001; Stage: *F_1,55_* = 187.237, *P* < 0.001), without a significant altitude × stage interaction (*F_1,55_* = 0.798, *P* = 0.375). Compared with the corresponding PH groups, liver mass was significantly decreased in both AH-HA and AH-LA groups (both *P* < 0.001), indicating reduced liver energy reserves after hibernation. Liver mass did not differ significantly between PH-HA and PH-LA (*P* = 0.082), whereas AH-LA had significantly lower liver mass than AH-HA (*P* < 0.001).

Unlike body mass and liver mass, HSI was significantly affected by altitude, stage and their interaction (Altitude: *F_1,52_* = 22.828, *P* < 0.001; Stage: *F_1,52_* = 766.336, P<0.001; Altitude × Stage: *F_1,52_* = 45.728, *P* < 0.001). Planned comparisons showed that HSI was significantly lower after hibernation in both populations (both *P* < 0.001). No significant difference was detected between PH-HA and PH-LA (*P* = 0.198), whereas AH-LA had significantly lower HSI than AH-HA (*P* < 0.001). The significant altitude × stage interaction indicates that the decrease in HSI differed between altitude populations, with a stronger reduction in the low-altitude population.

Metabolic rate was significantly affected by altitude and stage (Altitude: *F_1,19_* = 23.212, *P* < 0.001; Stage: *F_1,19_* = 75.894, *P* < 0.001), whereas the altitude × stage interaction was not significant (*F_1,19_* = 2.947, *P* = 0.102). Compared with the corresponding PH groups, metabolic rate increased significantly after hibernation in both populations (both *P* < 0.001). Between altitudes, PH-LA showed a significantly higher metabolic rate than PH-HA (*P* < 0.05), and AH-LA showed a significantly higher metabolic rate than AH-HA (*P* < 0.001).

Together, these results show that both high- and low-altitude populations experienced decreased body mass, liver mass and HSI, along with increased metabolic rate after hibernation. The significant altitude × stage interaction for HSI provides statistical support for altitude-dependent differences in relative liver energy depletion, whereas metabolic rate results indicate that the low-altitude population maintained a higher metabolic level before and after hibernation.

### 3.2 Changes in digestive tract length and small-intestinal histological structure

**Figure 3:**
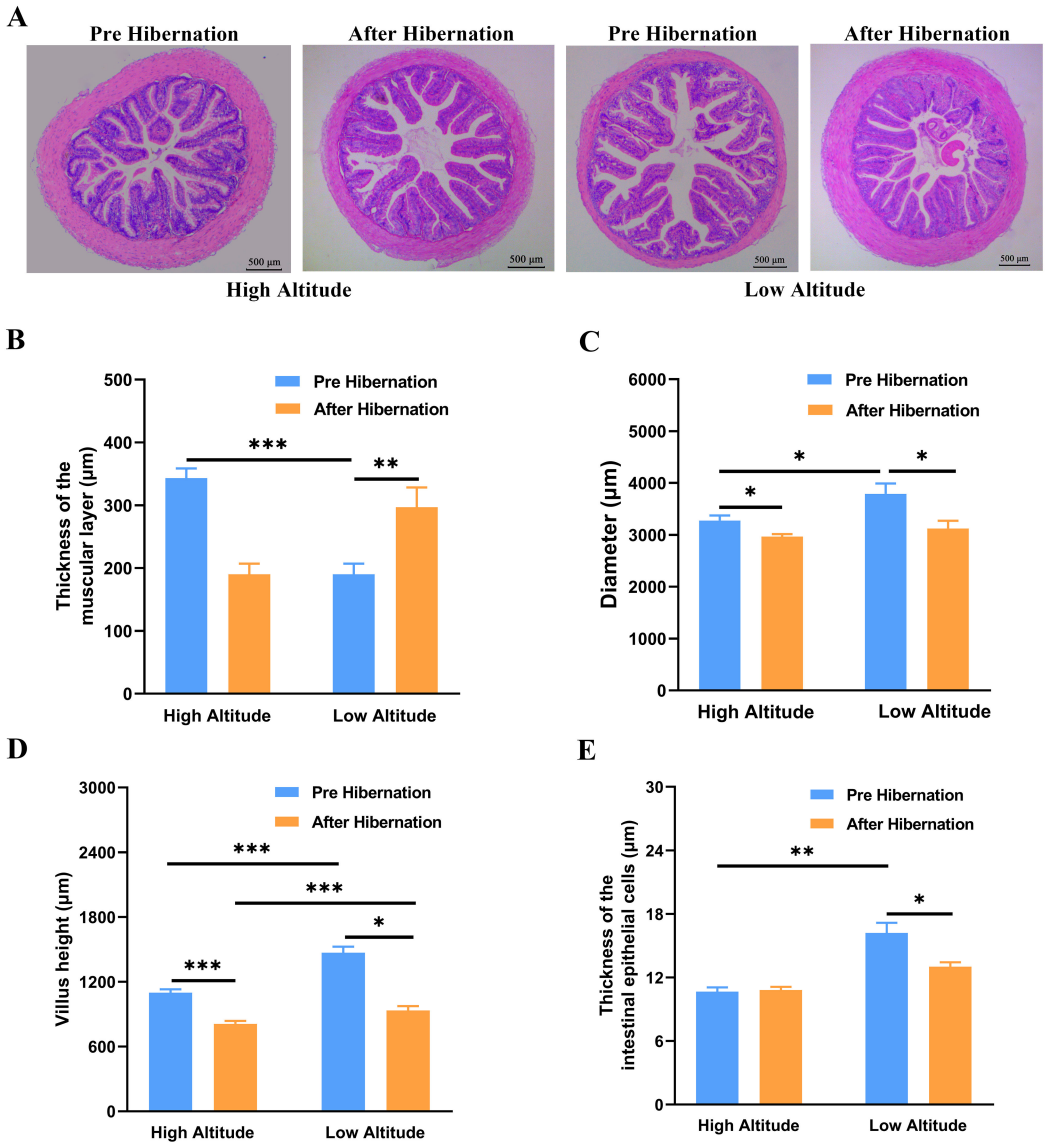
Changes in digestive system structure and small-intestinal histology before and after hibernation in high- and low-altitude populations of *Rana kukunoris*. **(A)** Representative hematoxylin and eosin-stained sections of small-intestinal tissues from the PH-HA, AH-HA, PH-LA, and AH-LA groups. Scale bar=500μm. Quantitative comparisons of small-intestinal histological parameters included lumen diameter **(B)**, muscularis thickness **(C)**, villus height **(D)**, and epithelial thickness **(E)**. Data are presented as mean ± SE. ^∗^ stands for *P* ≤ 0.05, ^∗∗^ stands for *P* ≤ 0.01, ^∗∗∗^ stands for *P* < 0.001.

To evaluate digestive system structural changes before and after hibernation, digestive tract length and small-intestinal histological parameters were analyzed using planned pairwise comparisons and two-way ANOVA (**Figure 3**, **Table 3**). Overall, both populations showed structural changes in the digestive system after hibernation, with more pronounced changes in the low-altitude population, including shortened digestive tract length, increased muscularis thickness, decreased villus height and reduced epithelial thickness.

**Table 3:** Two-way ANOVA results for digestive tract length and small-intestinal histological traits.

Variable	Effect	df	*F*	*P*
Digestive tract length	Altitude	1, 56	2.107	0.152
	Stage	1, 56	12.177	<0.001
	Altitude × Stage	1, 56	2.248	0.139
Lumen diameter	Altitude	1, 32	6.033	0.0197
	Stage	1, 32	12.718	0.00116
	Altitude × Stage	1, 32	1.730	0.198
Muscularis thickness	Altitude	1, 29	14.791	<0.001
	Stage	1, 29	2.672	0.113
	Altitude × Stage	1, 29	11.559	0.00198
Villus height	Altitude	1, 30	35.458	<0.001
	Stage	1, 30	92.046	<0.001
	Altitude × Stage	1, 30	8.380	0.00701
Epithelial thickness	Altitude	1, 29	39.450	<0.001
	Stage	1, 29	7.330	0.0112
	Altitude × Stage	1, 29	7.189	0.0120

Altitude indicates sampling altitude, Stage indicates hibernation stage, and Altitude × Stage indicates their interaction. P<0.05 was considered statistically significant.

Digestive tract length was significantly affected by stage (*F_1,56_* = 12.177, *P* < 0.001), whereas the effects of altitude (*F_1,56_* = 2.107, *P* = 0.152) and altitude × stage interaction (*F_1,56_* = 2.248, *P* = 0.139) were not significant. Planned comparisons showed no significant difference between PH-HA and AH-HA (*P* = 0.114), whereas AH-LA had a significantly shorter digestive tract than PH-LA (*P* < 0.01). No significant difference was detected between PH-HA and PH-LA (*P* = 0.978), whereas AH-LA had a significantly shorter digestive tract than AH-HA (*P* < 0.05).

H&E staining showed typical small-intestinal structures in all groups, including the intestinal lumen, mucosal folds, villi and peripheral muscularis layer (**Figure 3A**). Lumen diameter was significantly affected by altitude and stage, but not by the altitude × stage interaction (**Table 3**). Compared with the corresponding PH groups, lumen diameter decreased significantly after hibernation in both populations (both *P* < 0.05; **Figure 3B**). PH-LA had a significantly larger lumen diameter than PH-HA (*P* < 0.05), whereas no significant difference was detected between AH-HA and AH-LA (*P*>0.05).

Muscularis thickness was significantly affected by altitude and altitude × stage interaction (**Table 3**). Planned comparisons showed no significant difference between PH-HA and AH-HA (*P* = 0.147), whereas AH-LA had significantly greater muscularis thickness than PH-LA (*P* < 0.01; **Figure 3C**). PH-HA had significantly greater muscularis thickness than PH-LA before hibernation (*P* < 0.001), whereas no significant difference was detected between AH-HA and AH-LA after hibernation (*P*>0.05).

Villus height was significantly affected by altitude, stage and altitude × stage interaction (**Table 3**). Compared with the corresponding PH groups, villus height decreased significantly after hibernation in both AH-HA and AH-LA groups (both *P* < 0.001; **Figure 3D**). PH-LA showed significantly greater villus height than PH-HA (*P* < 0.001), and AH-LA showed significantly greater villus height than AH-HA (*P* < 0.05).

Epithelial thickness was significantly affected by altitude, stage and altitude × stage interaction (**Table 3**). Planned comparisons showed no significant difference between PH-HA and AH-HA (*P* = 0.797), whereas AH-LA had significantly lower epithelial thickness than PH-LA (*P* < 0.01; **Figure 3E**). In addition, PH-LA showed significantly greater epithelial thickness than PH-HA (*P* < 0.001), and AH-LA showed significantly greater epithelial thickness than AH-HA (*P* < 0.01).

In summary, small-intestinal structure changed to different degrees after hibernation in both altitude populations. The high-altitude population mainly showed decreased lumen diameter and villus height, while digestive tract length, muscularis thickness and epithelial thickness remained relatively stable. In contrast, the low-altitude population showed more pronounced structural remodeling, including shortened digestive tract length, reduced lumen diameter, increased muscularis thickness, decreased villus height and reduced epithelial thickness. Significant altitude × stage interactions in muscularis thickness, villus height and epithelial thickness further indicate different patterns of small-intestinal structural remodeling between altitude populations.

### 3.3 Metagenomic analysis of small-intestinal contents

#### 3.3.1 Overview of metagenomic sequencing and annotation

Metagenomic sequencing was performed on small-intestinal content samples from the PH-HA, AH-HA, PH-LA and AH-LA groups. After quality control and filtering, a total of 158,663,981,178 bp of clean data and 1,055,740,436 clean reads were obtained from 16 samples. Assembly generated 5,932,007 contigs, with an average N50 of 2,935 bp. A total of 7,906,099 ORFs were predicted, with the longest ORF reaching 109,527 bp. These results indicate that high-quality metagenomic data were obtained for subsequent analyses of taxonomic composition, microbial diversity and functional annotation.

#### 3.3.2 Alpha diversity of gut microbiota

**Figure 4:**
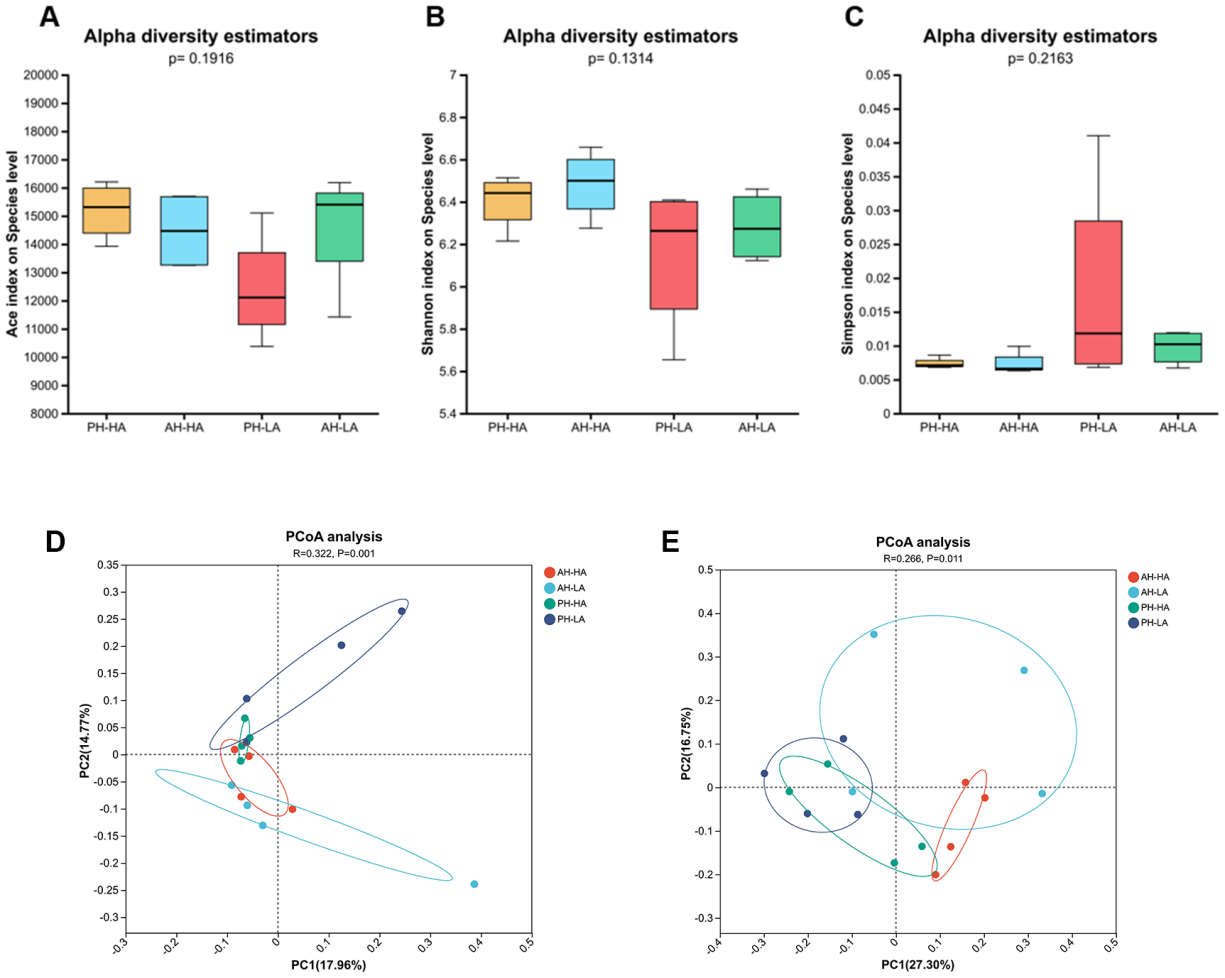
Alpha and beta diversity of small-intestinal microbiota in high- and low-altitude populations of *Rana kukunoris* before and after hibernation. Alpha diversity was evaluated using the ACE index **(A)**, Shannon index **(B)**, and Simpson index **(C)**. Beta diversity was visualized by principal coordinate analysis (PCoA) based on Binary Jaccard distance **(D)** and Bray–Curtis distance **(E)**.

ACE, Shannon and Simpson indices were used to evaluate alpha diversity of small-intestinal microbiota among the PH-HA, AH-HA, PH-LA and AH-LA groups. No significant differences were detected among the four groups for the ACE index (χ²=4.743, df=3, *P* = 0.1916; **Figure 4A**), Shannon index (χ²=5.625, df=3, *P* = 0.1314; **Figure 4B**) or Simpson index ((χ²=4.456, df=3,*P* = 0.2163; **Figure 4C**).

In terms of trends, the ACE index was relatively lower in PH-LA and relatively higher in PH-HA and AH-LA. The Shannon index was slightly higher in AH-HA and relatively lower in PH-LA and AH-LA. The Simpson index showed greater variation in PH-LA, whereas the other groups showed broadly similar distributions. However, the distributions largely overlapped among groups, and none of these differences reached statistical significance. Overall, microbial richness and evenness in small-intestinal contents remained relatively stable before and after hibernation, suggesting that hibernation stage and altitude did not significantly alter alpha diversity.

#### 3.3.3 Beta diversity of gut microbiota

**Figure 5:**
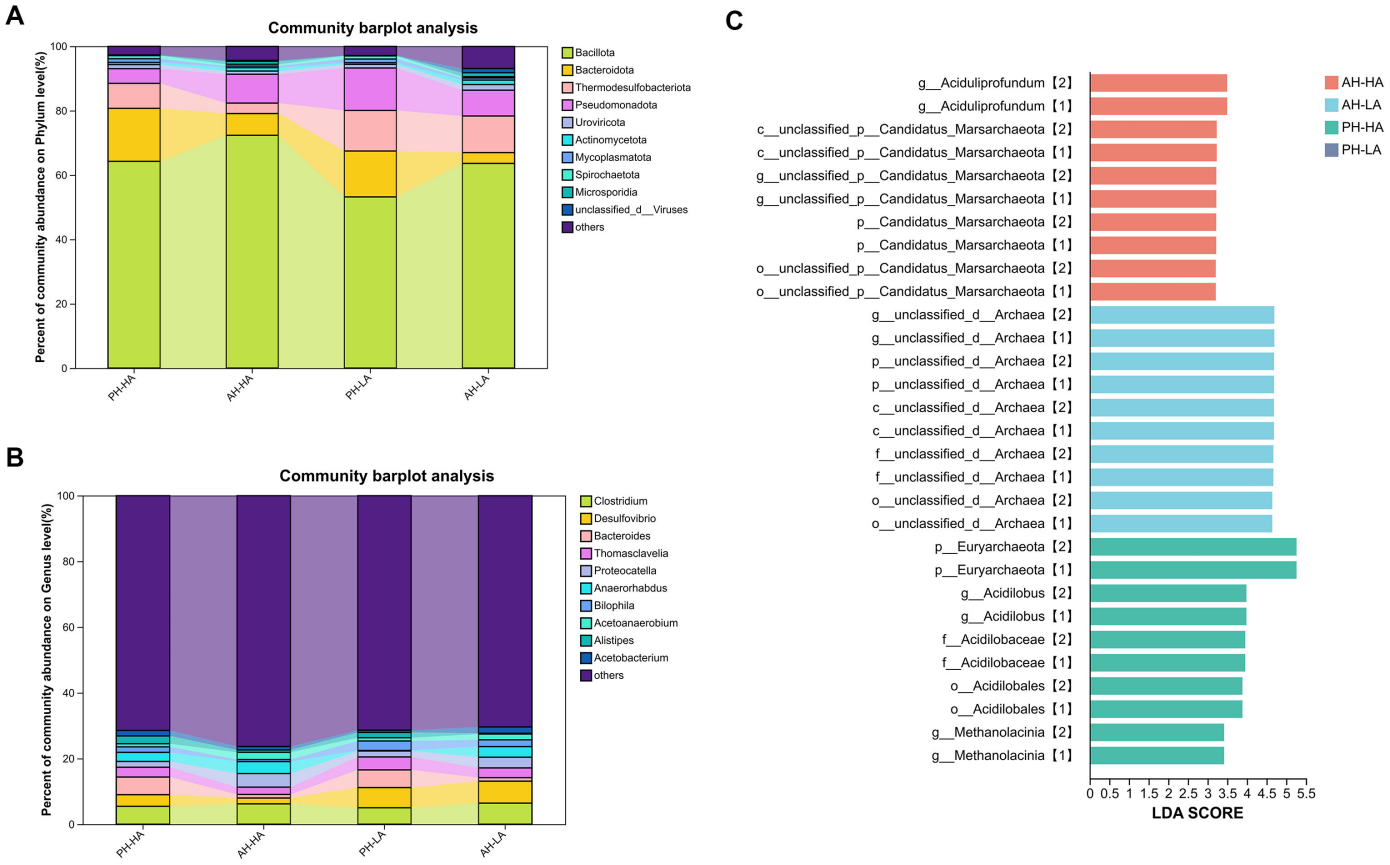
Microbial community composition and differentially enriched taxa in small-intestinal contents of *Rana kukunoris*. **(A)** Relative abundance of dominant microbial phyla in the PH-HA, AH-HA, PH-LA, and AH-LA groups. **(B)** Relative abundance of dominant microbial genera among groups. **(C)** Differentially enriched taxa identified by LEfSe analysis. Bar length represents the linear discriminant analysis (LDA) score, and different colors indicate taxa enriched in different groups, the suffixes [1] and [2] after taxon names are software-generated labels used to distinguish duplicated taxonomic names or taxonomic nodes with the same label in the LEfSe output.

PCoA based on Binary Jaccard and Bray–Curtis distance matrices showed significant differences in microbial community structure among PH-HA, AH-HA, PH-LA and AH-LA groups (**Figure 4D**). In the Binary Jaccard analysis, PC1 and PC2 explained 17.96% and 14.77% of the total variation, respectively, and ANOSIM indicated significant differences among groups (R = 0.322, *P* = 0.001; **Figure 4E**). The samples showed partial separation in the ordination space, with low-altitude-related samples tending to separate from the other groups, whereas PH-HA and AH-HA partially overlapped. PCoA based on Bray–Curtis distance also showed significant differences among groups (R = 0.266, *P* = 0.011; **Figure 5**), with PC1 and PC2 explaining 27.30% and 16.75% of the total variation, respectively. Similar to the Binary Jaccard results, the four groups showed partial separation in the ordination space. Overall, although alpha diversity did not differ significantly among groups, beta diversity analysis showed clear differentiation in microbial community structure, indicating that hibernation stage and altitude mainly affected community composition rather than within-sample richness or evenness.

#### 3.3.4 Gut microbial community composition

To explicitly characterize the dominant/core microbial communities associated with altitude and hibernation stage, we compared the major taxa shared across the four groups at the phylum and genus levels. At the phylum level (**Figure 5A**), Bacillota, Bacteroidota, Thermodesulfobacteriota and Pseudomonadota were the dominant phyla across the PH-HA, AH-HA, PH-LA and AH-LA groups, indicating that these taxa constituted the major microbial framework of the small-intestinal microbiota in *Rana kukunoris*. Other detected phyla included Uroviricota, Verrucomicrobiota, Mycoplasmatota, Spirochaetota, Actinomycetota and Euryarchaeota. The relative abundances of Bacillota, Bacteroidota, Thermodesulfobacteriota and Pseudomonadota were 64.24%, 16.64%, 7.75% and 4.57% in PH-HA; 72.33%, 6.76%, 3.25% and 8.96% in AH-HA; 53.19%, 14.26%, 12.60% and 13.19% in PH-LA; and 63.60%, 3.36%, 11.37% and 8.02% in AH-LA, respectively. Bacillota dominated all groups and increased after hibernation in both altitude populations, whereas Bacteroidota decreased after hibernation in both populations. These changes suggest a stage-related restructuring of the dominant phylum-level community during post-hibernation recovery. In contrast, Thermodesulfobacteriota maintained relatively high abundance in the low-altitude population, whereas Pseudomonadota increased after hibernation in the high-altitude population but decreased after hibernation in the low-altitude population, indicating that some microbial taxa may respond differently to hibernation stage depending on altitude background.

At the genus level (**Figure 5B**), the relatively abundant genera included *Clostridium, Desulfovibrio, Bacteroides, Proteocatella, Anaerorhabdus, Bilophila, Alistipes, Mucinivorans* and *Breznakia*. Among these genera, *Clostridium*, *Desulfovibrio*, *Bacteroides* and *Proteocatella* were representative dominant taxa showing clear group-level variation. Their relative abundances were 5.46%, 3.54%, 5.36% and 1.76% in PH-HA; 6.21%, 1.78%, 1.08% and 4.12% in AH-HA; 5.01%, 3.54%, 5.36% and 1.81% in PH-LA; and 6.43%, 6.68%, 1.06% and 3.25% in AH-LA, respectively.

Overall, the dominant genus-level community were broadly similar among groups, but the relative abundances of key genera differed according to altitude and hibernation stage. Clostridium increased after hibernation in both altitude populations, whereas Bacteroides decreased. Proteocatella was also higher after hibernation than before hibernation. In addition, Desulfovibrio showed relatively high abundance in AH-LA, indicating possible enrichment of this genus after hibernation in the low-altitude population. Taken together with the alpha and beta diversity results, these findings indicate that post-hibernation recovery did not markedly alter alpha diversity but was associated with restructuring of dominant microbial taxa and community composition at the group level.

#### 3.3.5 LEfSe analysis of differentially enriched microbial taxa

LEfSe analysis identified differentially enriched microbial taxa among groups (**Figure 5C**). In the AH-HA group, Aciduliprofundum and Candidatus_Marsarchaeota-related taxa, including phylum-level and unclassified class-, order- and genus-level taxa, were relatively enriched. In the AH-LA group, unclassified archaeal taxa (unclassified_d:Archaea) were enriched at multiple taxonomic levels, including phylum, class, order, family and genus. In the PH-HA group, Euryarchaeota, Acidilobales, Acidilobaceae, Acidilobus and Methanolacinia were relatively enriched. In contrast, no clearly enriched biomarker taxa were detected in the PH-LA group.

Overall, the differentially enriched taxa identified by LEfSe were mainly archaeal taxa, indicating differences in small-intestinal microbial composition among altitude populations and hibernation stages, with distinct archaeal enrichment patterns after hibernation.

#### 3.3.6 Functional annotation of gut microbiota

**Figure 6:**
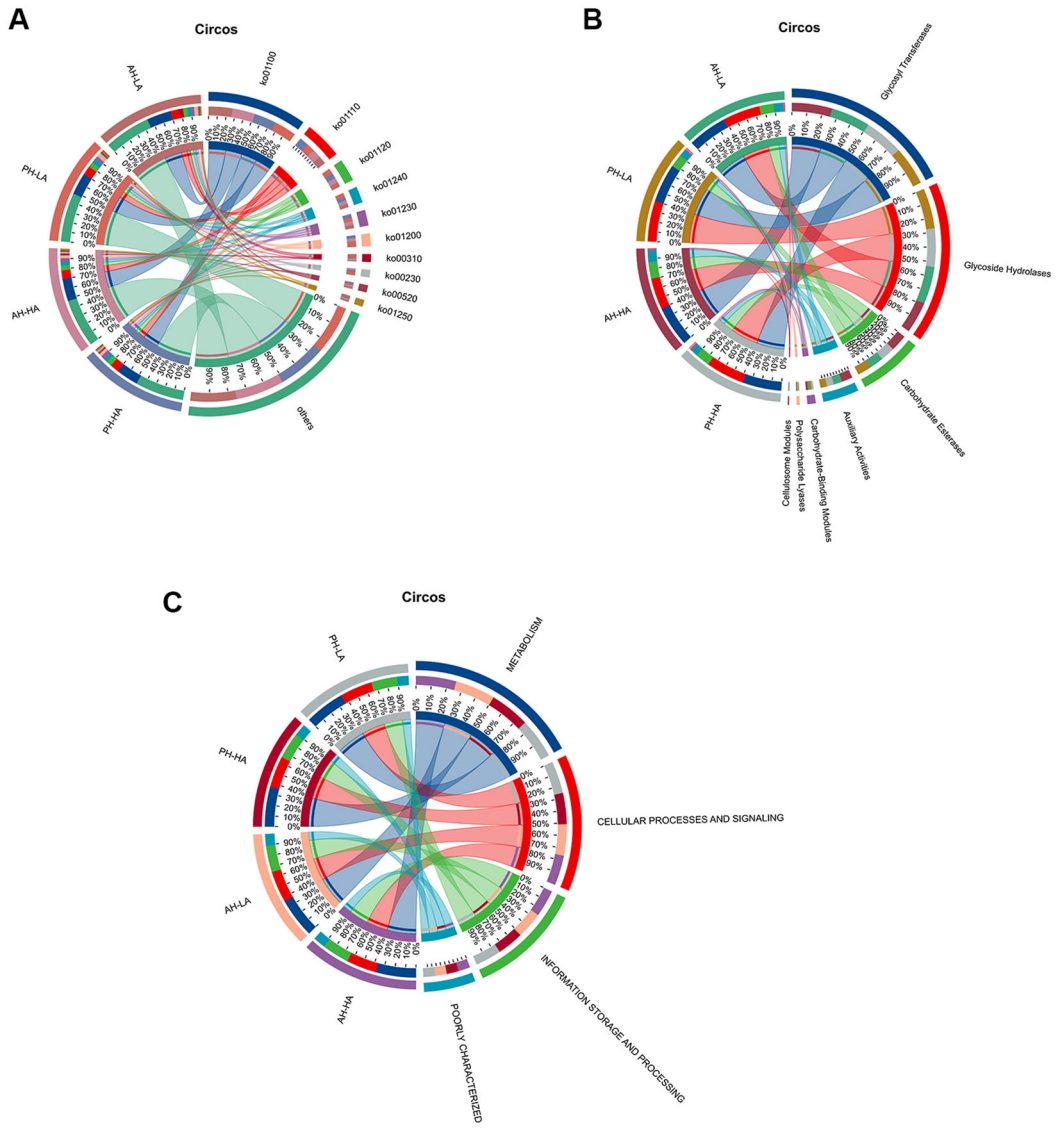
Functional annotation of small-intestinal microbiota in high- and low-altitude populations of *Rana kukunoris* before and after hibernation. **(A)** COG functional classification of non-redundant genes. **(B)** KEGG pathway annotation of microbial genes. **(C)** CAZy functional classification of carbohydrate-active enzyme genes. Functional profiles were compared among the PH-HA, AH-HA, PH-LA, and AH-LA groups to evaluate microbial functional potential before and after hibernation.

To further assess the functional potential of small-intestinal microbiota before and after hibernation in high- and low-altitude populations, non-redundant genes were annotated using the COG, KEGG and CAZy databases (**Figure 6**).

At the COG functional category level(**Figure 6A**), the overall distribution of functional annotations was similar among groups. Annotated genes were mainly assigned to Metabolism, Cellular processes and signaling, Information storage and processing, and poorly characterized. Among these categories, Metabolism and Cellular processes and signaling accounted for relatively high proportions, indicating that the microbial functional potential in small-intestinal contents was mainly associated with metabolic activities, cellular processes and signaling. At the KEGG level (**Figure 6B**), the annotated genes in all groups were mainly associated with metabolism-related pathways. ko01100 (Metabolic pathways) was the dominant pathway. In addition, ko01110 (Biosynthesis of secondary metabolites), ko01120 (Microbial metabolism in diverse environments), ko01230 (Biosynthesis of amino acids), ko01240 (Biosynthesis of cofactors) and ko01200 (Carbon metabolism) accounted for relatively high proportions. Overall, KEGG pathway composition was broadly similar among groups, although the relative contributions of major metabolic pathways varied among groups. At the CAZy level(**Figure 6C**), carbohydrate-active enzyme genes were mainly assigned to Glycosyl Transferases (GTs), Glycoside Hydrolases (GHs), Carbohydrate Esterases (CEs), Auxiliary Activities (AAs), Polysaccharide Lyases (PLs) and Carbohydrate-Binding Modules (CBMs). GTs and GHs were the dominant enzyme classes, followed by CEs, whereas AAs, PLs and CBMs contributed relatively lower proportions. These results indicate that small-intestinal microbiota in *R. kukunoris* have functional potential for carbohydrate synthesis, modification and degradation.

Together, COG, KEGG and CAZy functional annotations showed that although microbial community composition was restructured to some extent before and after hibernation and between altitude populations, the overall microbial functional potential remained centered on metabolism-related processes, particularly energy metabolism, nutrient transformation and carbohydrate utilization

## 4 Discussion

The present study compared high- and low-altitude populations of *R. kukunoris* before and after hibernation, with a particular focus on post-hibernation recovery, by integrating host energy status, whole-animal metabolic rate, digestive system structure and metagenomic profiles of small-intestinal contents. After hibernation, both populations showed decreased body mass, liver mass and HSI, along with increased metabolic rate, indicating a transition from energy depletion during hibernation to metabolic reactivation during recovery. At the digestive system level, digestive tract length remained relatively stable in the high-altitude population, and small-intestinal changes were mainly characterized by decreased lumen diameter and villus height. In contrast, the low-altitude population showed more pronounced digestive tract shortening, increased muscularis thickness, decreased villus height and reduced epithelial thickness. Metagenomic analysis further showed that alpha diversity did not differ significantly among groups, whereas beta diversity, dominant microbial taxa and functional potential were altered. Overall, these results suggest that post-hibernation recovery in *R. kukunoris* is not limited to a single physiological process but involves group-level parallel responses in host energy status, digestive system structure and gut microbiota.

### 4.1 Post-hibernation frogs are in a transitional state from energy depletion to metabolic recovery

Hibernation is a typical low-metabolic and energy-saving process based on reduced activity, decreased energy demand and continuous mobilization of endogenous energy reserves (Storey and Storey, 1990; 2007). In frogs, this low-metabolic state is reflected not only at the whole-animal level but also at the tissue level. For example, the aerobic capacity of skeletal muscle is markedly reduced during hibernation (St‐Pierre and Boutilier, 2001). Hibernation is also accompanied by pronounced suppression of immune function and gradual recovery after emergence (Cooper et al., 1992). More broadly, prolonged fasting and metabolic depression are important strategies that allow animals to survive food scarcity, and decreases in body mass and HSI can serve as important phenotypic indicators of energy reserve depletion (McCue, 2010).

In the present study, body mass, liver mass and HSI were significantly lower in AH-HA and AH-LA than in the corresponding pre-hibernation groups. This indicates that both high- and low-altitude populations experienced obvious energy depletion after hibernation. At the same time, metabolic rate increased significantly after hibernation in both populations, suggesting that frogs had transitioned from a low-metabolic overwintering state to a phase of metabolic reactivation during recovery. This pattern is consistent with the general sequence of “metabolic depression during hibernation followed by metabolic recovery after emergence” in hibernating animals (Storey and Storey, 1990; 2007).

Notably, although body mass and liver mass were both significantly affected by altitude and stage, their altitude × stage interactions were not significant. These two traits therefore mainly reflect general post-hibernation energy depletion and absolute differences between populations. In contrast, HSI was significantly affected by altitude, stage and their interaction, indicating that the magnitude of relative liver energy reserve depletion differed between altitude populations. Specifically, AH-LA showed significantly lower HSI than AH-HA, suggesting a stronger reduction in relative liver reserves in the low-altitude population after hibernation. Metabolic rate was also higher in PH-LA and AH-LA than in the corresponding high-altitude groups, indicating that the low-altitude population maintained a higher overall metabolic level before and after hibernation. However, because the altitude × stage interaction for metabolic rate was not significant, this result should be interpreted as a higher metabolic level in the low-altitude population rather than a significantly greater increase in metabolic rate after hibernation.

Previous studies on high-altitude amphibians have shown that adaptation to high-altitude environments can be reflected not only at genomic and transcriptomic levels but also in energy metabolism, antioxidant defense and physiological homeostasis (Muir et al., 2014; Yang et al., 2016; Niu et al., 2022; Chen et al., 2023; Niu et al., 2025; Tang et al., 2026). Therefore, our results suggest that the high-altitude population did not avoid post-hibernation energy depletion, but showed a relatively conservative pattern of liver reserve reduction and metabolic activation under the same seasonal transition. In contrast, the low-altitude population exhibited stronger relative liver energy depletion and a higher metabolic level, which may indicate greater energetic costs during post-hibernation recovery.

### 4.2 Digestive system structural changes indicate stronger seasonal plasticity in the low-altitude population

The digestive system is a key organ system for restoring feeding, rebuilding energy balance and compensating for overwintering energy depletion after hibernation. Its morphology and histological organization can reflect digestive and absorptive capacity as well as organ plasticity. Previous studies have shown that the intestines of amphibians and reptiles exhibit pronounced reversible regulation in response to fasting, refeeding and dormancy, including changes in intestinal mass, epithelial structure, digestive enzyme activity and nutrient transport capacity (Secor, 2005a, 2005; Tamaoki et al., 2016). Thus, digestive tract length and small-intestinal histological parameters can serve as important phenotypic indicators of digestive system plasticity before and after hibernation.

In this study, digestive tract length was mainly affected by hibernation stage, whereas the effects of altitude and the altitude × stage interaction were not significant. Planned comparisons showed that digestive tract length was significantly shorter in AH-LA than in PH-LA, whereas no significant difference was observed between PH-HA and AH-HA. This suggests a more pronounced shortening of the digestive tract after hibernation in the low-altitude population, although the absence of a significant interaction indicates that altitude dependence should be interpreted cautiously.

Small-intestinal histological traits provided stronger evidence for different structural responses between populations. Lumen diameter was affected by altitude and stage, but the interaction was not significant, indicating that both populations showed a decrease in lumen diameter after hibernation. Muscularis thickness, villus height and epithelial thickness all showed significant altitude × stage interactions, indicating that patterns of small-intestinal structural change differed between altitude populations. After hibernation, the high-altitude population mainly showed decreased lumen diameter and villus height, while digestive tract length, muscularis thickness and epithelial thickness remained relatively stable. In contrast, the low-altitude population showed digestive tract shortening, reduced lumen diameter, increased muscularis thickness, decreased villus height and reduced epithelial thickness.

From a physiological perspective, hibernation and prolonged fasting may reduce the maintenance costs of the digestive system, whereas post-hibernation recovery requires restoration of intestinal motility, digestion and absorption (Secor, 2005b; Kurtz et al., 2021). The increased muscularis thickness in the low-altitude population may be related to enhanced intestinal motility or tissue repair during recovery. Decreased villus height and epithelial thickness suggest marked remodeling of absorptive structures. By contrast, although the high-altitude population also showed changes in lumen diameter and villus height, the overall magnitude of remodeling was lower. This may indicate that the high-altitude population maintains a more stable digestive structure to reduce the energetic cost of organ reconstruction after hibernation, which is consistent with the need to conserve recovery costs under cold, hypoxic and short-season high-altitude conditions.

### 4.3 Gut microbiome restructuring reflects changes in community structure and functional potential during post-hibernation recovery

In this study, ACE, Shannon and Simpson indices did not differ significantly among the four groups, whereas PCoA based on Binary Jaccard and Bray–Curtis distances showed significant differentiation in microbial community structure. This indicates that the main microbial changes before and after hibernation were not large shifts in within-sample richness or evenness, but rather rearrangements in community structure and the relative abundances of dominant taxa. Similar seasonal restructuring has been reported in amphibians during artificial hibernation, natural overwintering and spring emergence [8,9,12]. It has also been observed in hibernating mammals such as ground squirrels, in which gut microbiota are restructured across the hibernation cycle in association with fasting, mucus utilization, bile metabolism and changes in host metabolic status (Carey et al., 2013; Dill‐McFarland et al., 2014; Carey and Assadi-Porter, 2017).

In terms of seasonal changes, Bacillota increased after hibernation in both altitude populations, whereas Bacteroidota decreased. At the genus level, Clostridium and Proteocatella increased after hibernation, whereas Bacteroides decreased. These patterns suggest that both populations underwent similar directional restructuring of dominant microbial taxa during post-hibernation recovery. Bacillota and Clostridium include many anaerobic fermentation-related taxa, and their increased relative abundance may reflect enhanced potential for substrate transformation and fermentation during recovery. In contrast, the decrease in Bacteroides may indicate that substrate availability, mucus utilization or feeding recovery had not fully returned to the pre-hibernation state during early recovery. The Bacillota (formerly Firmicutes)/Bacteroidota ratio is often discussed in relation to host energy harvest and nutrient substrate utilization, but this relationship is not simply linear and is more appropriately interpreted as a change in microbial metabolic potential and substrate utilization strategy (Turnbaugh et al., 2006).

Altitude-related differences were also evident in the microbiome data. The low-altitude population showed more pronounced changes in microbial composition after hibernation. Desulfovibrio showed relatively high abundance in AH-LA, suggesting that anaerobic redox processes and sulfur-containing substrate metabolism may be more strongly altered in the small intestine of the low-altitude population after hibernation. Previous studies have shown that strict anaerobes such as *Bilophila wadsworthia* can utilize dietary or host-derived organic sulfur substrates and produce H_2_S, which has biphasic effects in the gut environment, participating in physiological regulation but potentially causing toxicity or inflammation when excessive (Feng et al., 2017; Burrichter et al., 2021; Buret et al., 2022). Thus, changes in taxa such as Desulfovibrio and Bilophila in the low-altitude population may be associated with stronger intestinal remodeling and recovery-related metabolic changes. LEfSe analysis further showed that differentially enriched taxa were mainly archaeal, including Aciduliprofundum and Candidatus_Marsarchaeota-related taxa in AH-HA and unclassified archaeal taxa in AH-LA. These findings suggest that different altitude populations may have distinct anaerobic metabolic features and gut microbial restructuring patterns before and after hibernation.

Functional annotation further showed that COG, KEGG and CAZy profiles were centered on metabolism-related functions, particularly carbon metabolism, amino acid metabolism, cofactor biosynthesis and carbohydrate-active enzyme functions. Although community composition changed, microbial functional potential remained mainly associated with nutrient transformation, energy acquisition and homeostatic processes (Claus et al., 2011; Holmes et al., 2012; Nicholson et al., 2012; Tremaroli and Bäckhed, 2012; Sommer and Bäckhed, 2013). This pattern may be explained by microbial functional redundancy, whereby different taxa can perform overlapping metabolic functions and taxonomic shifts do not necessarily lead to complete changes in functional structure (Louca et al., 2018). The relatively high proportions of GTs, GHs and CEs in the CAZy annotation indicate that small-intestinal microbiota have functional potential for complex carbohydrate synthesis, modification and degradation. Previous studies have shown that gut bacteria can contribute to host nutrient recovery and energy metabolism through complex polysaccharide degradation and short-chain fatty acid production (Pryde et al., 2002; Flint et al., 2008; Louis and Flint, 2016; Schwalm and Groisman, 2017). Therefore, our results suggest that, during post-hibernation recovery, gut microbiota may maintain a metabolism-centered functional framework while undergoing taxonomic restructuring, potentially supporting nutrient transformation and energy balance.

### 4.4 High- and low-altitude populations may adopt different post-hibernation recovery strategies

Integrating host energy status, digestive system structure and metagenomic results, high- and low-altitude populations of *R. kukunoris* appear to exhibit different response patterns during post-hibernation recovery. Across the four groups, Bacillota, Bacteroidota, Thermodesulfobacteriota and Pseudomonadota represented the dominant phylum-level microbial framework, whereas *Clostridium*, *Desulfovibrio*, *Bacteroides* and *Proteocatella* were representative dominant genera showing group-level variation. These dominant/core microbial communities were broadly shared across altitude populations and hibernation stages, but their relative abundances differed, indicating that post-hibernation recovery was associated mainly with restructuring of the existing intestinal microbial community rather than complete replacement of the dominant microbial framework. The high-altitude population showed decreased body mass, liver mass and HSI and increased metabolic rate after hibernation, but digestive tract length, muscularis thickness and epithelial thickness remained relatively stable. Small-intestinal remodeling was mainly reflected by decreased lumen diameter and villus height. In addition, PH-HA and AH-HA partially overlapped in the beta diversity ordination space, suggesting relatively smaller changes in microbial community structure. In contrast, the low-altitude population showed lower body mass, liver mass and HSI, higher metabolic rate, more pronounced digestive tract shortening and stronger small-intestinal remodeling, along with increases in Bacillota, *Clostridium* and *Proteocatella* and decreases in Bacteroidota and *Bacteroides*. The relatively high abundance of Desulfovibrio in AH-LA further suggested that some microbial taxa may be specifically enriched after hibernation in the low-altitude population. Together, these patterns suggest that the low-altitude population may rely on stronger metabolic activation, digestive structural remodeling and microbial community restructuring to support the recovery of feeding, digestion and nutrient absorption after hibernation.

From a statistical perspective, the significant altitude × stage interactions for HSI, muscularis thickness, villus height and epithelial thickness provide key evidence supporting different post-hibernation recovery patterns between altitude populations. Although body mass, liver mass, metabolic rate and digestive tract length showed significant stage effects or altitude effects, their interactions were not significant. Therefore, these traits are more suitable for describing general post-hibernation changes and absolute differences between populations rather than serving as independent evidence for significantly different magnitudes of change.

Based on these findings, the high-altitude population may adopt a recovery strategy characterized by relatively conservative metabolic activation and lower-magnitude digestive structural remodeling, which may help reduce recovery costs under cold environments with short activity seasons. In contrast, the low-altitude population may rely on a higher metabolic level and stronger tissue plasticity to rapidly rebuild feeding, digestion and absorption. This difference may be related to long-term altitude-associated environmental selection. Previous studies have shown that high-altitude frogs exhibit adaptive changes at genomic, transcriptomic, physiological, biochemical and metabolic levels [1–5]. Thus, low temperature, hypoxia and short activity seasons at high altitudes may favor low-cost restoration of essential functions after hibernation rather than extensive organ reconstruction, whereas the relatively milder overwintering environment at lower altitudes may allow stronger tissue remodeling and metabolic activation during recovery.

Overall, this study shows that post-hibernation recovery in *R. kukunoris* involves parallel responses in energy reserve depletion, metabolic recovery, digestive system structural adjustment and gut microbiome restructuring. The high-altitude population exhibited a relatively conservative recovery pattern with lower-magnitude structural remodeling, whereas the low-altitude population showed stronger relative liver energy depletion, higher metabolic level and more pronounced small-intestinal plasticity. Gut microbiota were restructured in community composition and functional potential while maintaining a metabolism-centered functional framework, suggesting a possible role in nutrient transformation and energy balance during post-hibernation recovery. Because this study is mainly based on group-level comparisons, future studies integrating individual-matched sampling and key metabolite measurements will be useful for further testing the links among host phenotypes, intestinal structure and microbial function. These findings provide new evidence for understanding post-hibernation recovery strategies and host–gut microbiome parallel responses in amphibians inhabiting cold high-altitude environments. In addition, the number of metagenomic samples per group was limited; therefore, the microbiome results should be interpreted as group-level patterns of microbial community composition and functional potential. Future studies with larger sample sizes and paired host–microbiome datasets are needed to further validate individual-level associations among host physiology, intestinal structure and gut microbial functions.

## 5 Conclusion

This study demonstrates that post-hibernation recovery in the plateau frog *R. kukunoris* involves cross-level parallel responses in host energy status, digestive system structure and gut microbiota. After hibernation, both high- and low-altitude populations showed decreases in body mass, liver mass and hepatosomatic index, together with an increase in whole-animal metabolic rate, indicating a transition from energy reserve depletion to metabolic recovery. Compared with the high-altitude population, the low-altitude population exhibited stronger relative liver energy depletion, a higher metabolic level and greater small-intestinal structural plasticity, whereas the high-altitude population showed a relatively conservative recovery pattern with lower-magnitude digestive remodeling. The microbiota of small-intestinal contents underwent community restructuring while maintaining relatively stable alpha diversity and a metabolism-centered functional potential related to nutrient transformation and carbohydrate utilization. Overall, these findings suggest that high- and low-altitude populations of *R. kukunoris* may adopt different physiological recovery strategies after hibernation and provide new evidence for understanding seasonal adaptation and host–gut microbiome parallel responses in amphibians inhabiting cold environments.

## Data Availability

The raw shotgun metagenomic sequencing data generated in this study have been deposited in the NCBI Sequence Read Archive under BioProject accession number PRJNA1480428.
